# Sclerotherapy Verus Propranolol After a Variceal Bleed

**DOI:** 10.1155/1993/89427

**Published:** 1993

**Authors:** Gregory Van Stiegmann

**Affiliations:** University of Colorado and Denver Veterans Affairs Hospital Box C-313, 4200 East 9th Av. Denver CO 80262 USA


operation[11]. Until surgeons are more alert to a preoperative or peroperative diagnosis and until the limits of radical surgery are defined the further treatment of gallbladder carcinoma invading perimuscular connective tissue but not serosa must remain an open question.

### REFERENCES

1. Paraskevopoulos, J.A., Dennison, A. and Johnson, A.G. (1991) Primary carcinoma of the gallbladder. *HPB-Surg.*, **4**, 277–289
2. Jones, R.S. (1990) Carcinoma of the gallbladder. *Surg. Clin. North Am.*, **70**, 1419–1428
3. Nadlar, L.H. and McSherry, C.K. (1992) Carcinoma of the gallbladder: review of the literature and report on 56 cases at the Beth Israel Medical Center. *Mount Sinai J. Med.*, **59**, 47–52
4. Bergdahl, L. (1980) Gallbladder carcinoma first diagnosed by microscopic examination of gallbladders removed for presumed benign disease. *Ann. Surg.*, **191**, 19–22
5. de-Aretxabala, X., Roa, I., Araya, J.C., Burgos, L., Flores, P., Huenchullan, I. and Miyazaki, I. (1990) Operative findings in patients with early forms of gallbladder cancer. *Br. J. Surg.*, **77**, 291–293
6. de-Aretxabala, X., Roa, I., Burgos, L., Araya, J.C., Fonseca, L., Wistuba, I. and Flores, P. (1992) Gallbladder cancer in Chile. A report on 54 potentially resectable tumors. *Cancer*, **69**, 60–65
7. Houry, S., Schlienger, M., Huguier, M., Lecaine, F., Perine, F. and Lugier, A. (1989) Gallbladder carcinoma. Role of radiation therapy. *Br. J. Surg.*, **76**, 448–450
8. Busse, P.M., Cady, B., Bothe, A., Jenkins, R., McDermott, W.D., Steele, G. and Stone, M.D. (1991) Intraoperative radiation therapy for carcinoma of the gallbladder. *World J. Surg.*, **15**, 352–356
9. Mimura, M., Takakura, M., Kim, H., Hamazaki, K., Tsuge, H. and Ochiai, Y. (1991) Block resection of the hepatoduodenal ligament for carcinoma of the bile duct and gallbladder. *Hepatogastroenterology*, **38**, 561–567
10. Matzumoto, Y., Fujii, E., Aoyama, H., Yamamoto, M., Sugahara, K. and Suda, K. (1992) The surgical treatment of primary carcinoma of the gallbladder based on the histologic analysis of 48 surgical specimens. *Am. J. Surg.*, **163**, 239–245
11. Pezet, D., Fondrinier, E., Rotman, N., Guy, L., Lemesle, P., Lointier, P. and Chipponi, J. (1992) Parietal seeding of carcinoma of the gallbladder after laparoscopic cholecystectomy. *Br. J. Surg.*, **79**, 230

A.N. Kingsnorth
Senior Lecturer in Surgery
Department of Surgery
The University of Liverpool
P O Box 147
Liverpool L69 3BX
United Kingdom

### SCLEROTHERAPY VERUS PROPRANOLOL AFTER A VARICEAL BLEED

ABSTRACT

*Dasarathy, S., Dwivedi, M., Bhargava, D.K., Sundaram, K.R. and Ramachandran, K. (1992) A prospective randomized trial comparing repeated endoscopic sclerotherapy and propranolol in decompensated (Child B and C) cirrhotic patients. Hepatology, 16, 89–95.*

**A prospective randomized study was conducted to compare the efficacy of long-term endoscopic sclerotherapy versus propranolol in Child class B and C patients with variceal bleeds within the 30 days before the study. Forty-five and 46 patients were randomized to receive sclerotherapy and propranolol, respectively, after preentry stratification for Child scores. Sclero-therapy was administered with 1% polidocanol at 10-day intervals until obliteration of varices was achieved. Propranolol was administered to achieve a reduction in resting pulse rate of 25%. Rebleeding occurred in 19 patients undergoing sclerotherapy and in 31 receiving propranolol ($p < 0.05$). The number of episodes of rebleeding was higher ($p < 0.05$) in the propranolol group ($n = 64$) than in the sclerotherapy group ($n = 35$). The mean bleeding risk factor, number of hospitalizations for rebleeding and blood transfusion requirement were also significantly higher in the propranolol-treated patients. The median bleed-free period was more than 36 mo in the sclero-therapy group and 2.5 mo in the propranolol group ($p < 0.01$). The median survival time was significantly longer in the sclerotherapy group (> 36 mo) than in the propranolol group (> 24 mo). We conclude that in decompensated cirrhotic patients, long-term endoscopic sclerotherapy is superior to propranolol in preventing rebleeding and improving survival. (Hepatology, 1992; 16: 89–94).**

**PAPER DISCUSSION**

KEY WORDS: Oesophageal varices, sclerotherapy, propranolol therapy

Dasarathy and colleagues have contributed the sixth published trial comparing the beta adrenergic antagonist propranolol with serial endoscopic sclerotherapy for prevention of recurrent bleeding from esophageal varices[1]. In contrast to some of the previous studies, only Child-Pugh class B and C patients were entered. In further contrast to all but one of the previous trials, a significant reduction in both the incidence of recurrent bleeding from esophageal varices and in mortality was observed in patients who had endoscopic treatment.

Table 1 summarizes information from the current trial and the five previously published studies which compared endoscopic sclerotherapy with propranolol[2–6]. Alexandrino *et al.* found a significant reduction in the incidence of recurrent hemorhage from esophageal varices in sclerotherapy treated patients as well as a trend toward improved survival. The latter was also observed by Westaby *et al.* and Flieg *et al.* On the other hand, Dollet *et al.* demonstrated a trend toward less rebleeding in patients treated with propranolol but had identical survival in each treatment arm while Rossi *et al.* found no differences at all.

Can meaningful conclusions be drawn from six trials with such disparate results? The wide variation in outcomes between these trials suggests several explanations: patients in different trials were dissimiliar; endoscopic sclerotherapy was performed more effectively in some trials; or the titration of propranolol and/or compliance with taking the drug was better in some trials.

The latter seems least likely since all of the trials treated patients with propranolol to reduce resting pulse rates from 20 to 25% and compliance with taking the drug was good. On the other hand, entry criteria, sclerotherapy technique, and sclerotherapy treatment intervals varied widely across these studies. The majority of patients in the five previously published trials had alcohol induced cirrhosis in

**Table 1** Results of six prospective randomized trials which compared serial sclerotherapy with propranolol for prevention of recurrent bleeding from esophageal varices. S = sclerotherapy, P = propranolol, % Rebled = proportion of patients who experienced one or more episodes of recurrent hemorrhage from esophagael varices, % Child C = total proportion of Child-Pugh class C patients in both arms of trial, % EtOH = proportion of alcoholic cirrhotics in both arms of trial (*$p$>0.05)

| *Author* | *# Patients* | *Therapy* | *%Rebled* | *%Survival* | *%Child C* | *%EtOH* |
|---|---|---|---|---|---|---|
| Flieg | 36 | S | 28% | 91% | 30% | 82% |
| (4) | 34 | P | 29% | 85% | | |
| Alexandrino | 31 | S | 33%* | 69% | 0 | 80% |
| (2) | 34 | P | 75%* | 54% | | |
| Westaby | 56 | S | 45% | 66% | 0 | 55% |
| (6) | 52 | P | 54% | 55% | | |
| Rossi | 26 | S | 50% | 80% | 38% | 100% |
| (5) | 27 | P | 47% | 79% | | |
| Dollet | 28 | S | 64% | 51% | 27% | 94% |
| (3) | 27 | P | 44% | 53% | | |
| Dasarathy | 45 | S | 42%* | 78%* | 34% | 27% |
| (1) | 46 | P | 67%* | 59%* | | |

contrast to the Desarathy trial in which 73% had non-alcoholic liver disease. Two of the previous trials completely excluded Child-Pugh class C patients and selection was so careful in one that 83 percent of those admitted with bleeding varices were eliminated[2]. Entry criteria for the Desarathy study were less exclusive. Specifically, patients were not excluded, as in other studies, if transfusions for the index bleed exceeded a fixed number (e.g. 6 or 10) of units of blood and all patients were required to have grade four (large) esophageal varices and endoscopic signs indicating a high risk of recurrent hemorrhage. Patients were also required to be hemodynamically stable for a period of only 24 hours prior to randomization. Other trials varied the time interval from the index bleed to randomization: intervals ranged up to greater than 15 days[2]. Methods of sclerotherapy varied considerably among the studies and included both intravariceal (five trials) and paravariceal injections administered repetitively at intervals as short as two to four days and as long as three weeks until varices were eradicated. Patients who did experience recurrent bleeding (both treatment groups) were treated medically (vasoconstrictor plus balloon tamponade if needed) in five of the trials (including Desarathy *et al.*) and shunt surgery or emergency sclerotherapy (the latter held by many to be the optimal treatment for acute bleeding) was employed if bleeding persisted after removal of the tamponade device. Only one trial employed emergency sclerotherapy at the time of the endoscopic diagnosis of recurrent bleeding from varices[6].

Is endoscopic sclerotherapy a better treatment than propranolol, particularly for patients with poorly compensated liver disease? Results from the three previous trials which included Child class C patients are inconclusive. More than 75 percent of the patients in these three studies were in Child-Pugh class B and C but none of these trials resulted in significant differences in outcome variables in favor of one treatment over the other. Two of the three examined the incidence of rebleeding and/or survival rates between cohorts when stratified by Child-Pugh class and found no differences, although the numbers of individual patients in each subset were small. These data, derived predominately from selected patients with alcoholic cirrhosis, support the conclusion that sclerotherapy, as employed in these studies, was neither better nor worse than propranolol in the groups studied.

What conclusions then can be drawn from the study of Desarathy *et al.* which convincingly demonstrates what all therapeutic endoscopists instinctively know—that swallowing an endoscope is better than swallowing a pill? It is generally agreed that propranolol may not be as effective for prevention of recurrent hemorrhage from esophageal varices in patients with advanced liver disease as it is in those with better preserved hepatic function. Results from the five previous sclerotherapy versus propranolol trials do not fully support this conclusion but the very design of two of them (Child-Pugh class C patients excluded) and the relatively stringent entry criteria for the remainder suggest that selection of patients to be treated with propranolol is very important. The etiology of cirrhosis also seems to play a role in the success of pharmacological therapy with propranolol. The patient with non-alcoholic cirrhosis appears to be at a disadvantage when treated with propranolol, particularly when compared to the patient with alcoholic cirrhosis who refrains from alcohol use. The findings of Dasarathy *et al.* are valuable because the patient who is best treated with propranolol as an alternative to endoscopic or operative therapy has yet to be precisely defined. Until results of this trial are confirmed and such definitions are well established, the use of propranolol should remain confined to carefully selected patients or those entered into controlled trials.

### REFERENCES

1. Dasarathy, S., Dwivedi, M., Bhargava, D., Sundaram, K. and Ramachandran, K. (1992) A prospective randomized trial comparing repeated endoscopic sclerotherapy and propranolol in decompensated (Child class B and C) cirrhotic patients. *Hepatology*, **16**, 89–94
2. Alexandrino, P.T., Martins Alves, M. and Correia, J.P. (1988) Propranolol or endoscopic sclerotherapy in the prevention of recurrent variceal bleeding. *J. Hepatology*, **7**, 175–185
3. Dollet, J., Champigneulle, B., Patris, A., Bigard, M. and Gaucher, P. (1988) Sclerotherapie endoscopique contre propranolol apres hemorragie par rupture varices oesophagiennes chez le cirrhotique: Resultats a 4 ans d'une etude randomisee. *Gastroenterol. Clin. Biol.*, **12**, 234–239
4. Flieg, F.E., Stange, E.F., Hunecke, R., *et al.* (1987) Prevention of recurrent bleeding in cirrhotics with recent variceal hemorrhage: Prospective, randomized comparison of propranolol and sclerotherapy. *Hepatology*, **7**, 355–361
5. Rossi, V., Cales, P., Burtin, P., *et al.* (1991) Prevention of recurrent variceal bleeding in alcoholic cirrhotic patients: prospective controlled trial of propranolol and sclerotherapy. *J. Hepatology*, **12**, 283–289
6. Westaby, D., Polson, R.J., Gimson, A.E.S., Hayes, P.C., Hayllar, K. and Williams, R. (1990) A controlled trial of oral propranolol compared with injection sclerotherapy for the long-term management of variceal bleeding. *Hepatology*, **11**, 353–359

Gregory Van Stiegmann
Associate Professor of Surgery
Chief Surgical Endoscopy
University of Colorado and
Denver Veterans Affairs Hospital
Box C-313, 4200 East 9th Av.
Denver, CO 80262, USA

